# Supplementary material for: Effect of Dexamethasone on Thermoresponsive Behavior of Poly(2-Oxazoline) Diblock Copolymers
Source: Polymers (Basel). 2021 Apr 21;13(9):1357. doi: 10.3390/polym13091357 (PMC8122420; doi:10.3390/polym13091357)
Supplement: Supplementary file 1 [file polymers-13-01357-s001.zip › polymers-1183628 - Supplementary Final.pdf]

polymers

MDPI

Supplementary Materials

Effect of Dexamethasone on Thermoresponsive Behavior of Poly(2-oxazoline) Diblock Copolymers

Monika Majerčíková [1], Peter Nádaždy [2], Dušan Chorvát [3], Leonid Satrapinskyy [4], Helena Valentová [5], Zuzana Kroneková [1], Peter Šiffalovič [2,6], Juraj Kronek [1,*] and Anna Zahoranová [7,*]

1 Department for Biomaterials Research, Polymer Institute of the Slovak Academy of Sciences, Dúbravská cesta 9, 845 41 Bratislava, Slovakia; majercikova.monika@savba.sk (M.M.); zuzana.kronekova@savba.sk (Z.K.); juraj.kronek@savba.sk (J.K.)
2 Institute of Physics of the Slovak Academy of Sciences, Dúbravská cesta 9, 845 11 Bratislava, Slovakia; peter.nadazdy@savba.sk (P.N.); peter.siffalovic@savba.sk (P.S.)
3 Department of Biophotonics, International Laser Centre, Ilkovičova 3, 841 04 Bratislava, Slovakia; Dusan.Chorvat@ilc.sk
4 Department of Experimental Physics, Faculty of Mathematics, Physics and Informatics, Comenius University, Mlynska Dolina, 842 48 Bratislava, Slovakia; leonid.satrapinskyy@fmph.uniba.sk
5 Faculty of Mathematics and Physics, Charles University, Ke Karlovu 3, 121 16 Prague 2, Czech Republic; helena.valentova@mff.cuni.cz
6 Centre for Advanced Material Application, Slovak Academy of Sciences, Dúbravská cesta 9, 845 11 Bratislava, Slovakia
7 Institute of Applied Synthetic Chemistry, Vienna University of Technology, Getreidemarkt 9/163MC, A-1060 Vienna, Austria; anna.zahoranova@tuwien.ac.at
* Correspondence: juraj.kronek@savba.sk (J.K.); anna.zahoranova@tuwien.ac.at (A.Z.)

Citation: Majerčíková, M.; Nádaždy, P.; Chorvát, D.; Satrapinskyy, L.; Valentová, H.; Kroneková, Z.; Šiffalovič, P.; Kronek, J.; Zahoranová, A. Effect of Dexamethasone on Thermoresponsive Behavior of Poly(2-oxazoline) Diblock Copolymers. *Polymers* **2021**, *13*, 1357. https://doi.org/10.3390/polym13091357

Academic Editor: Xiao Hu

Received: 30 March 2021
Accepted: 17 April 2021
Published: 21 April 2021

Publisher's Note: MDPI stays neutral with regard to jurisdictional claims in published maps and institutional affiliations.

Copyright: © 2021 by the authors. Licensee MDPI, Basel, Switzerland. This article is an open access article distributed under the terms and conditions of the Creative Commons Attribution (CC BY) license (http://creativecommons.org/licenses/by/4.0/).

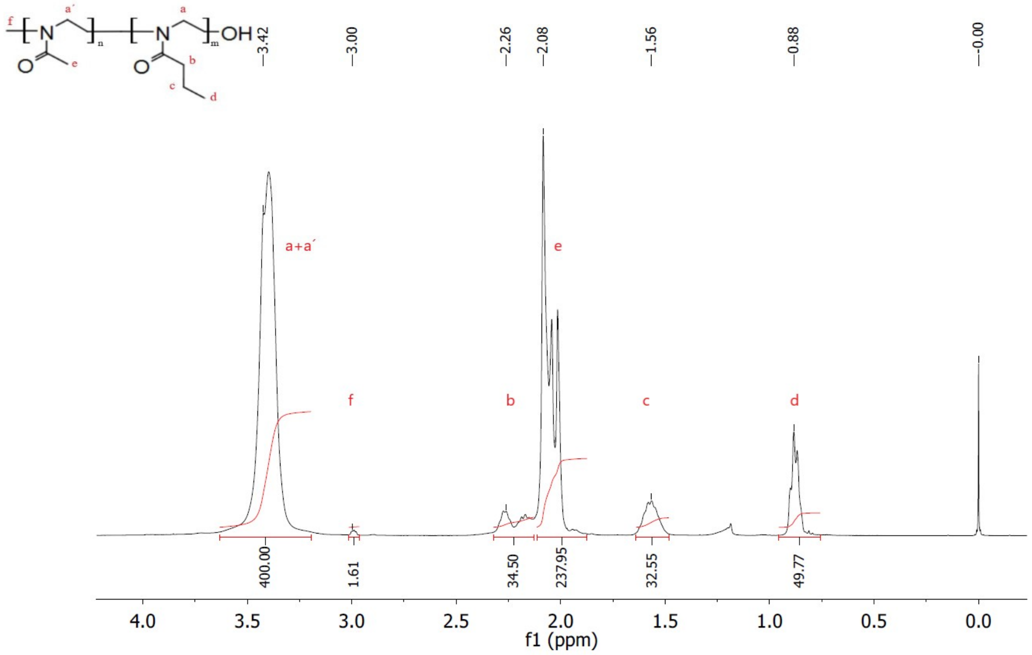

Figure S1. ^{1}H NMR spectrum of copolymer P1 (nPrOx$_{20}$:MeOx$_{80}$) in $CDCl_3$.

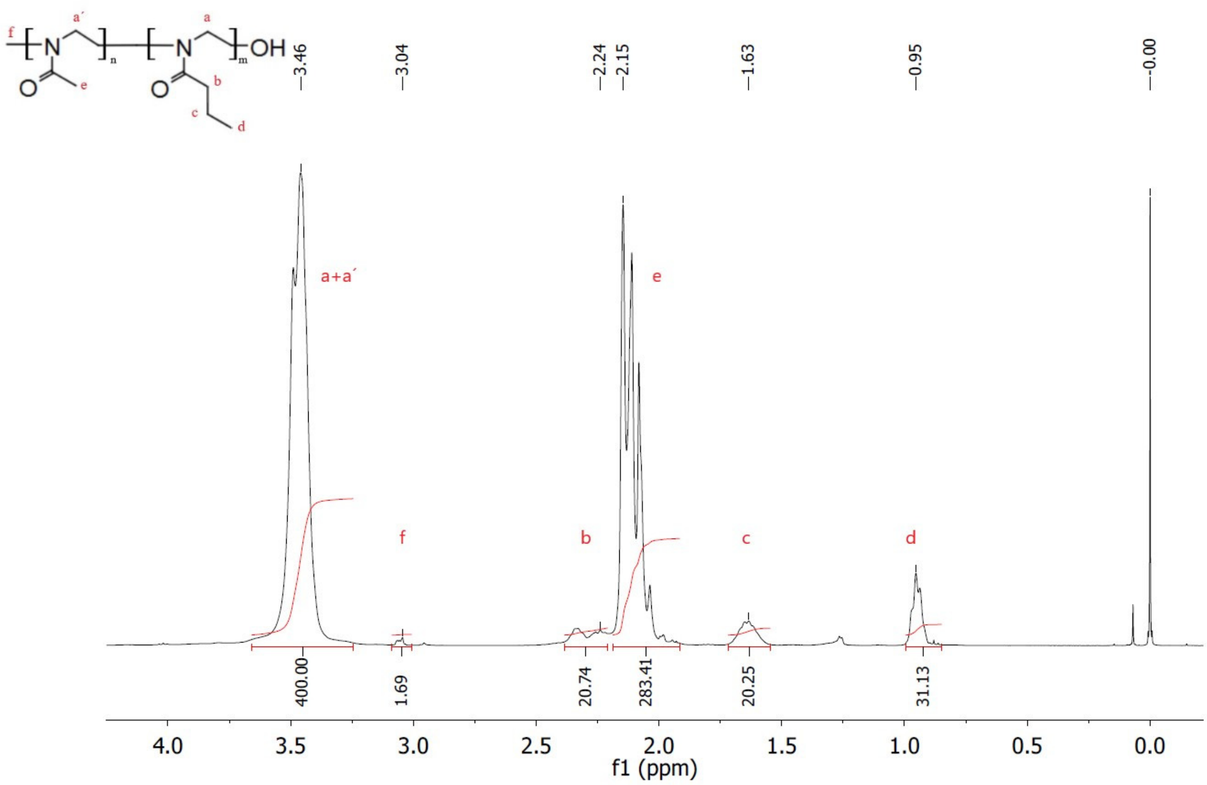

Figure S2. ^{1}H NMR spectrum of P2 (nPrOx$_{10}$:MeOx$_{90}$) in $CDCl_3$.

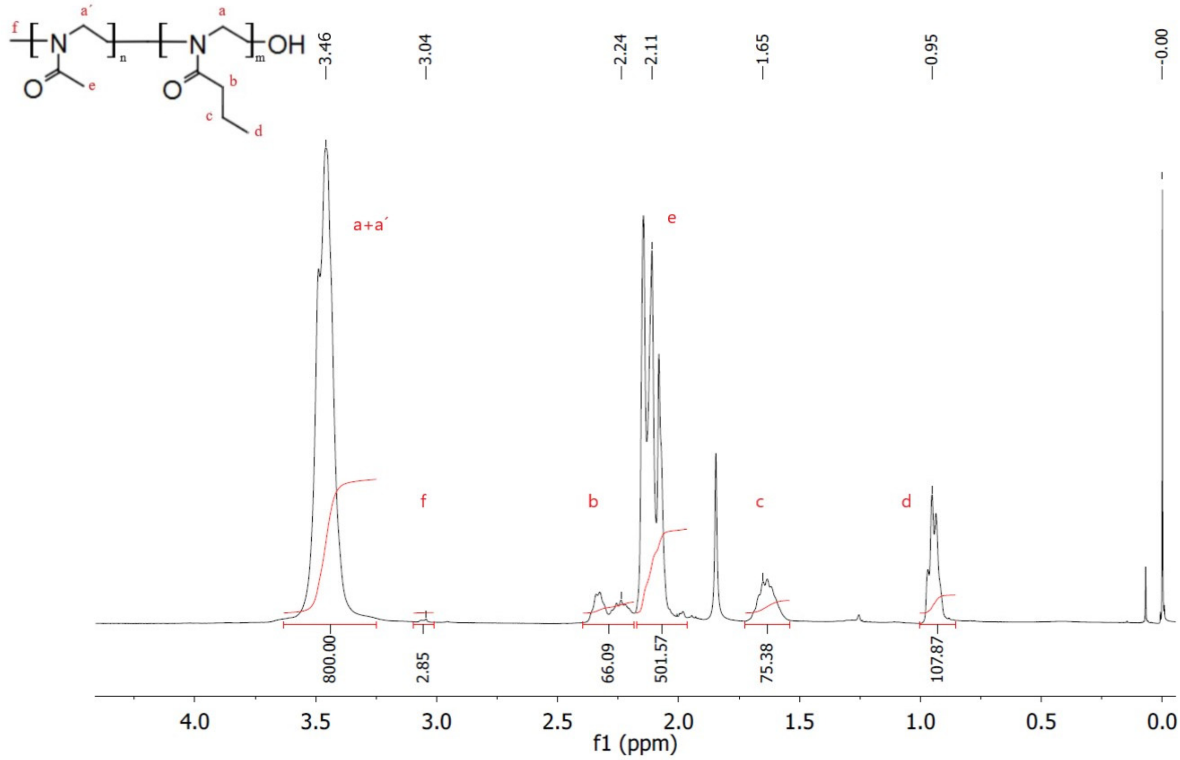

Figure S3. ^{1}H NMR spectrum of P3 (nPrOx$_{40}$:MeOx$_{160}$) in $CDCl_3$.

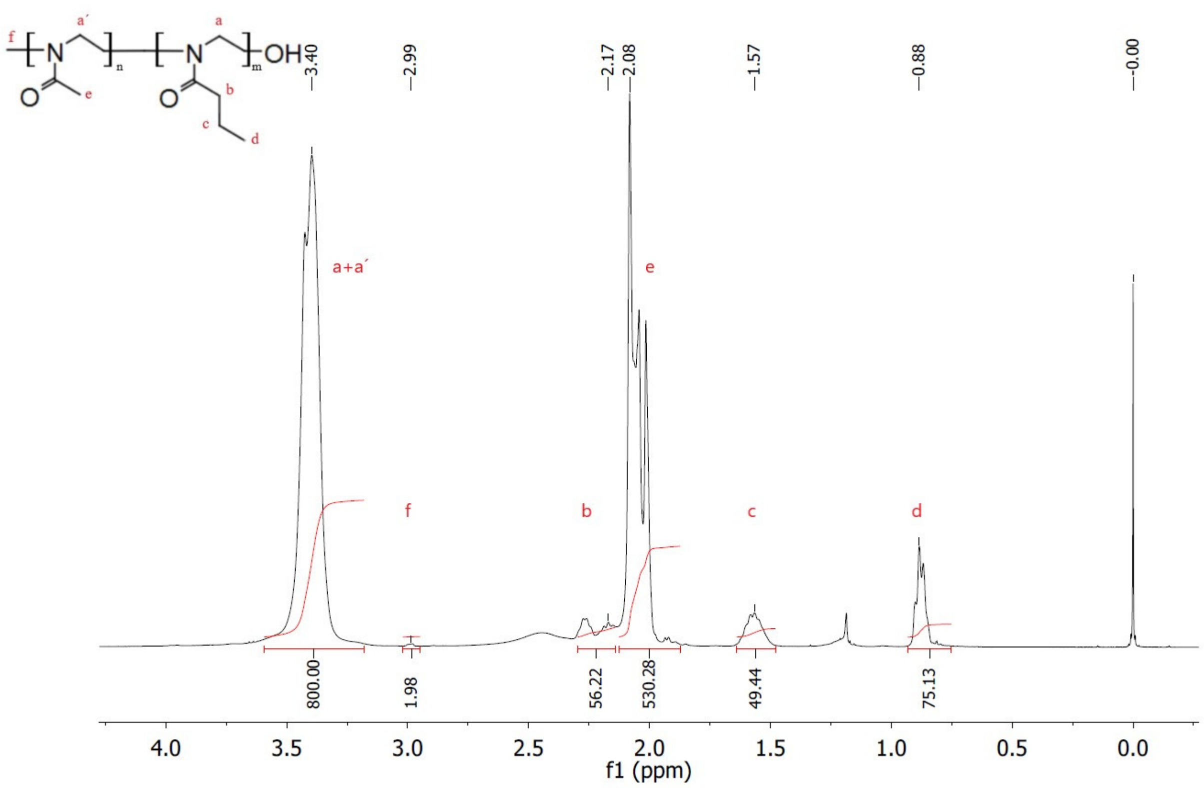

Figure S4. ^{1}H NMR spectrum of P4 (nPrOx$_{20}$:MeOx$_{180}$) in $CDCl_3$.

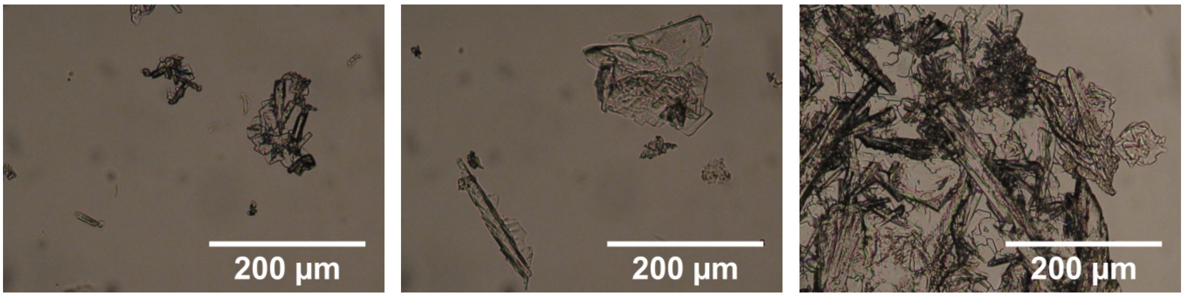

Figure S5. Optical microscope images of Dexa (2 mg·mL^{-1}) in PBS, prepared by dissolution in ethanol, evaporation and subsequent re-hydration, analogously to Dexa-copolymer samples.

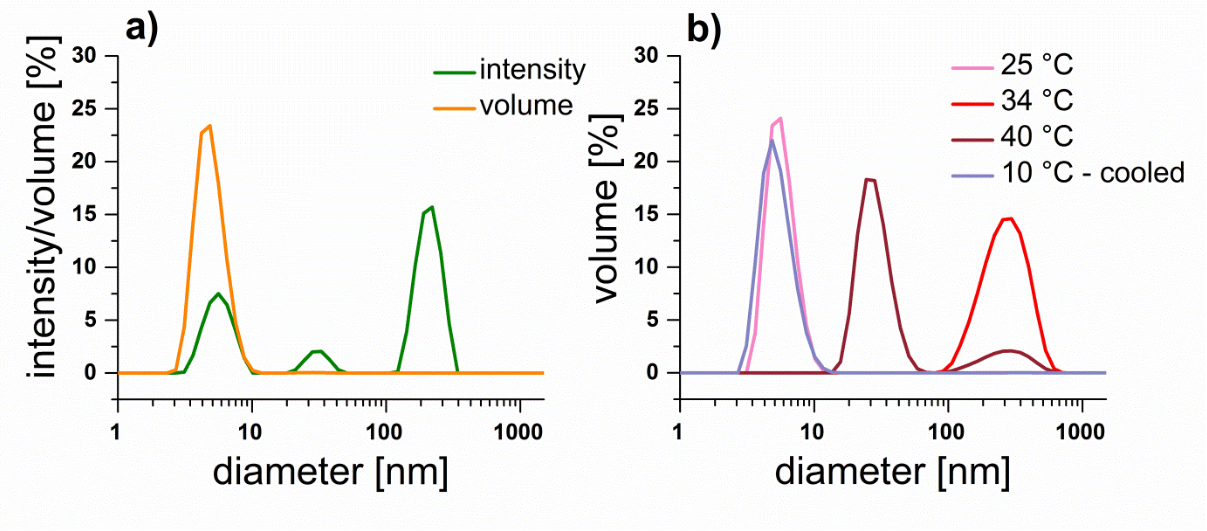

Figure S6. Comparison of intensity-weighted and volume-weighted size distribution of sample P2 with the concentration 10 mg.mL^{-1} without Dexa at 40°C in PBS measured with DLS (**a**). Sample P1 with the concentration 10 mg.mL^{-1} of copolymer and 0.5 mg.mL^{-1} Dexa at various temperatures (sample was cooled to 10°C after heating), volume-weighted size distribution from DLS is displayed (**b**).

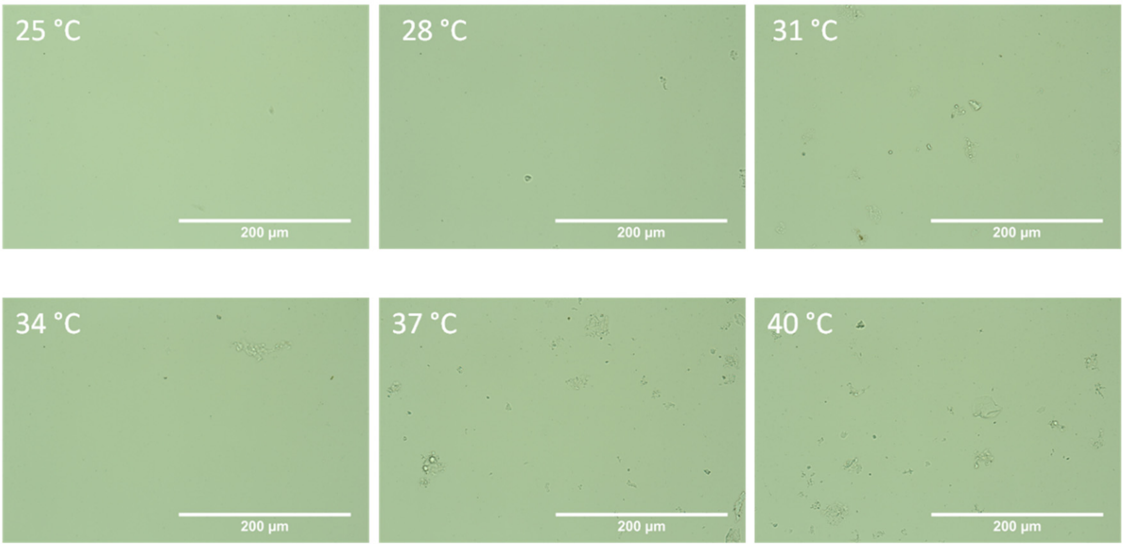

Figure S7. Optical images of P1 copolymer (10 mg mL^{-1}) without Dexa in distilled water visualized by an optical microscope with increasing temperature. No microparticles are formed**.**

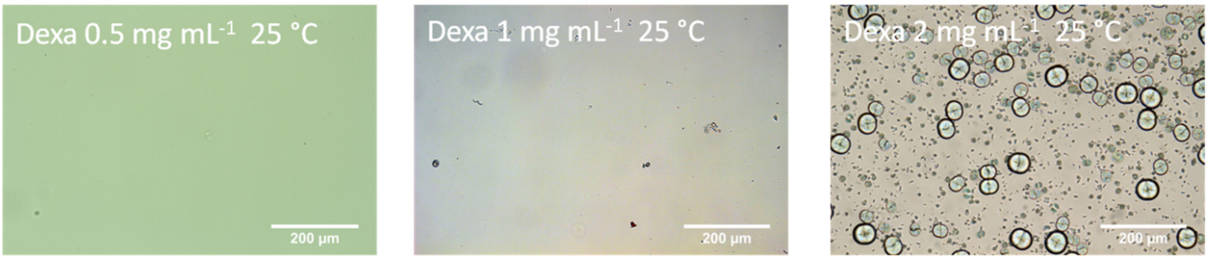

Figure S8. P1 copolymer (10 mg·mL^{-1}) with Dexa (0.5, 1, 2 mg·mL^{-1}) prepared by a thin-film method visualized by an optical microscope at 25 °C.

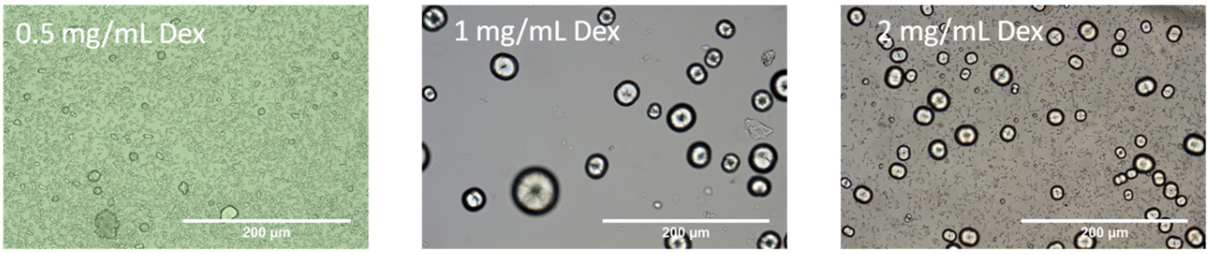

Figure S9. P1 copolymer (10 mg·mL^{-1}) with Dexa (0.5, 1, 2 mg·mL^{-1}) prepared by a thin-film method visualized by an optical microscope after 15 min cooling at 8 °C.

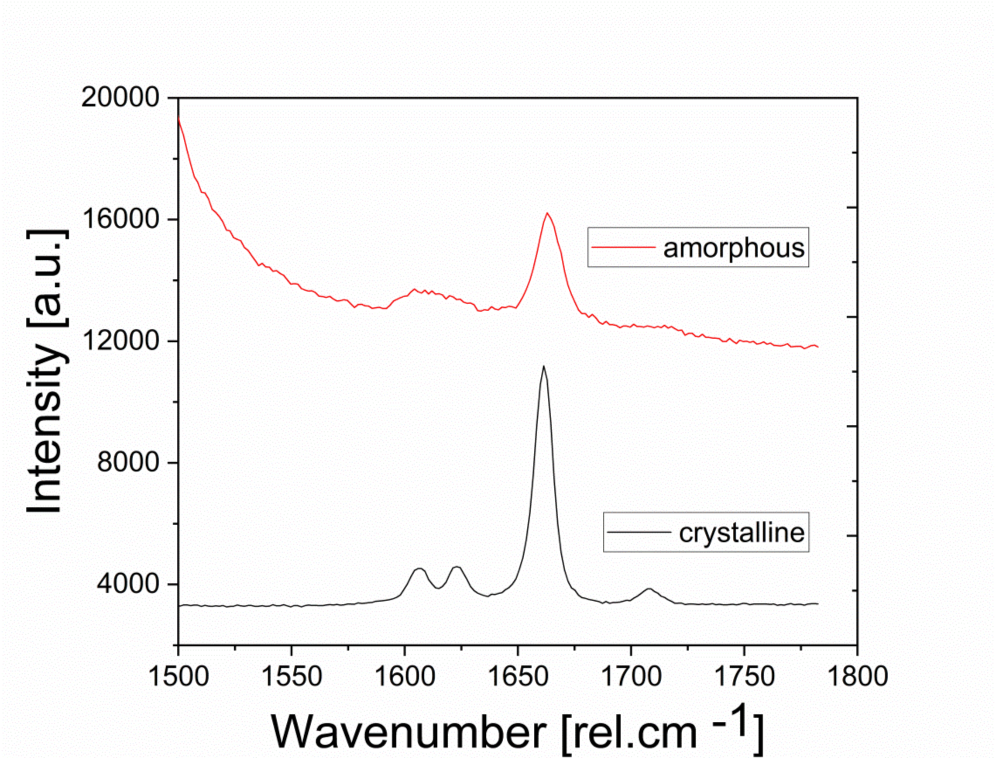

Figure S10. Raman spectra of the amorphous and crystalline forms of dexamethasone.
